# Vitamin D levels are associated with trait resilience but not depression in a general population sample

**DOI:** 10.1002/brb3.1884

**Published:** 2020-10-13

**Authors:** Jan Terock, Anke Hannemann, Deborah Janowitz, Jasmin Müller, Henry Völzke, Hans J. Grabe

**Affiliations:** ^1^ Department of Psychiatry and Psychotherapy University Medicine Greifswald Greifswald Germany; ^2^ Department of Psychiatry and Psychotherapy HELIOS Hanseklinikum Stralsund Stralsund Germany; ^3^ Institute of Clinical Chemistry and Laboratory Medicine University Medicine Greifswald Greifswald Germany; ^4^ Institute for Community Medicine University Medicine Greifswald Greifswald Germany; ^5^ German Center for Neurodegenerative Diseases (DZNE) Greifswald Germany

**Keywords:** depression, general population, resilience, rs4588, rs7041, vitamin D

## Abstract

**Introduction:**

Insufficient vitamin D levels were found to be related to various psychiatric disorders and particularly depression. The functional polymorphisms rs4588 and rs7041 of the vitamin D‐binding protein (also **g**roup‐specific **c**omponent or Gc) influence vitamin D level and activity. Resilience is considered the individual predisposition to maintain psychological functioning in the face of adversities. We sought to investigate whether associations of vitamin D levels and genotypes of rs4588 and rs7041 were associated with trait resilience and symptoms of depression.

**Methods:**

Serum levels of total 25(OH)D were measured in a general population sample (*n* = 1,908) of the Study of Health in Pomerania (SHIP‐1). The Resilience Scale‐25 (RS‐25) was applied to assess trait resilience. Lifetime depressive symptoms were assessed using the CID‐S, while current depressive symptoms were measured using the Beck Depression Inventory II (BDI‐II). Study participants were genotyped for rs4588 and rs7041.

**Results:**

Participants with vitamin D insufficiency had lower adjusted mean RS‐25 scores as compared to vitamin D replete subjects (*p* = .002). Linear regression analyses revealed a positive association between 25(OH)D and RS‐25 scores (*ß* = 2.782, *p* = .002). Additional adjustment for BDI‐II scores slightly attenuated this result (*ß* = 1.830 and *p* = .026). Symptoms of depression and the lifetime diagnosis of MDD were not significantly associated with vitamin D concentrations. rs4588 and rs7041 showed strong associations with vitamin D concentrations (both *p* < .001), but not RS‐25 scores.

**Conclusions:**

In contrast with previous studies, our findings do not provide evidence for a strong role of vitamin D in the psychopathology of depression. However, considering the role of trait resilience as a common protective factor to different psychiatric disorders, our results support the concept of low vitamin D as a general risk factor to stress‐related psychopathologies.

## Graphical abstract

We found that resilience, but not depression, was strongly associated with vitamin D levels in 1,908 subjects from the general population. The association with resilience remained stable after adjustment for depressive symptoms. Functional polymorphisms of the vitamin D‐binding protein gene (rs4588 and rs7041) were highly associated with vitamin D levels, but neither depression nor resilience.

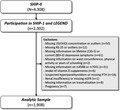

## 1 INTRODUCTION

Evidence from previous studies suggested that low vitamin D levels and vitamin D insufficiency are involved in the pathogenesis of various psychopathologies and particularly affective disorders: For example, Parker et al. (2017) reported in their review and meta‐analysis that vitamin D insufficiency was linked with depression and that vitamin D supplementation showed beneficial effects in depressed patients with vitamin D insufficiency (Parker et al., 2017). Also, vitamin D insufficiency was found to be associated with increased risk for anxiety disorders in animal and human studies (Armstrong et al., 2007; Huang et al., 2014; Kalueff et al., 2004). Other studies consistently showed strong associations of schizophrenia (Valipour et al., 2014) as well as bipolar disorder (Boerman et al., 2016) with low vitamin D levels. Finally, in a recent study of our working group, we demonstrated that vitamin D levels were negatively associated with occurrence of posttraumatic stress disorder (PTSD) (Terock et al., 2020). In addition, we found that two common functional polymorphisms of the vitamin D‐binding protein (rs4588 and rs7041) were linked to PTSD occurrence even after adjusting for vitamin D levels.

The term "resilience" was established to describe the psychological and biological predispositions helping to maintain a stable level of psychological functioning in the face of traumatic stress (Rutter, 1987). Different personality characteristics including secure attachment, sense of coherence and purpose in life, and self‐acceptance have been identified as factors which promote resilience (Wagnild & Young, 1993). Results from previous studies showing particularly negative associations of resilience with depression and PTSD suggest that resilience is a protective factor for stress‐related psychiatric disorders (Bensimon, 2012). More recent refinements proposed that resilience represents a dynamic personality trait with genetic, metabolic, and psychosocial determinants, which is flexible and can be influenced by therapeutic approaches (Bowes & Jaffee, 2013). Despite the converging results pointing at a role of vitamin D metabolism in the development of diverse psychiatric disorders, to our best knowledge, no study has investigated whether vitamin D levels and genotypes of the Gc gene were related to trait resilience.

Vitamin D can be obtained by dietary intake from plants (as vitamin D_2_ or ergocalciferol) and animal sources (as vitamin D_3_ or cholecalciferol) or generated in the body when sunlight reaches the skin, where ultraviolet B radiation is involved in the conversion of 7‐dehydrocholesterol to vitamin D_3_. In the next steps, ingested or autosynthesized vitamin D_3_ is converted in the liver to 25‐hydroxyvitamin D [25(OH)D]. 25(OH) D is the main circulating metabolite and reflects the long‐term vitamin D supply of the organism. Finally, 25(OH)D is metabolized into in the active form of 1,25‐dihydroxyvitamin D [1,25(OH)D] by the enzyme 1α‐hydroxylase primarily in the kidneys, but also in other tissues including the brain (Eyles et al., 2005).

More than 99% of the circulating vitamin D metabolites are bound to different proteins including albumin and chylomicrons. However, the majority is bound to vitamin D‐binding protein (VDBP, also known as “**g**roup‐specific **c**omponent,” Gc), which carries between 85% and 90% of total circulating 25(OH)D and 1,25(OH)D (Bikle et al., 1986; White & Cooke, 2000).

Plasma levels of the Gc protein and its affinity to vitamin D metabolites are influenced by two common functional polymorphisms in the Gc gene, rs4588 and rs7041 (Arnaud & Constans, 1993; Boutin et al., 1989). Given that only the free fraction of 1,25(OH)D can enter easily the intracellular compartment and exert its actions in target tissues (Bikle & Gee, 1989), it has been suggested that these polymorphisms influence vitamin D effects. The combined polymorphisms of rs4588 and rs7041 encode for three common protein isoforms (Gc1F, Gc1S, and Gc2). In populations of European ancestry, Gc1S represents the most abundant genotype, followed by Gc2 and finally Gc1F (Powe et al., 2013). Specifically, plasma concentrations of Gc and total vitamin D were found to be highest in homozygous carriers of the Gc1F or Gc1S isoforms, intermediate in Gc1‐2 genotypes, and lowest in homozygous subjects with the Gc2 phenotype (Lauridsen et al., 2005). Likewise, the variants of the VDBP were found to show differences in their affinity to both, 25(OH)D and 1,25(OH)D with a sequence of Gc1F > Gc1S > Gc2 (Arnaud & Constans, 1993).

In all, based on the previous findings showing associations of decreased vitamin D levels with depression and other psychopathologies and the role of resilience as a common protective factor to stress‐related psychiatric disorders, we hypothesized that (a) vitamin D levels are negatively associated with depressive symptoms as well as the lifetime diagnosis of major depressive disorder (MDD) and (b) positively associated with trait resilience. Moreover, we (c) expected that genotypes of the Gc gene which are linked to enhanced gene expression and higher vitamin D levels are also associated with enhanced resilience and lower depressive symptoms.

## 2 MATERIALS AND METHODS

### 2.1 Study population

**FIGURE 1:**
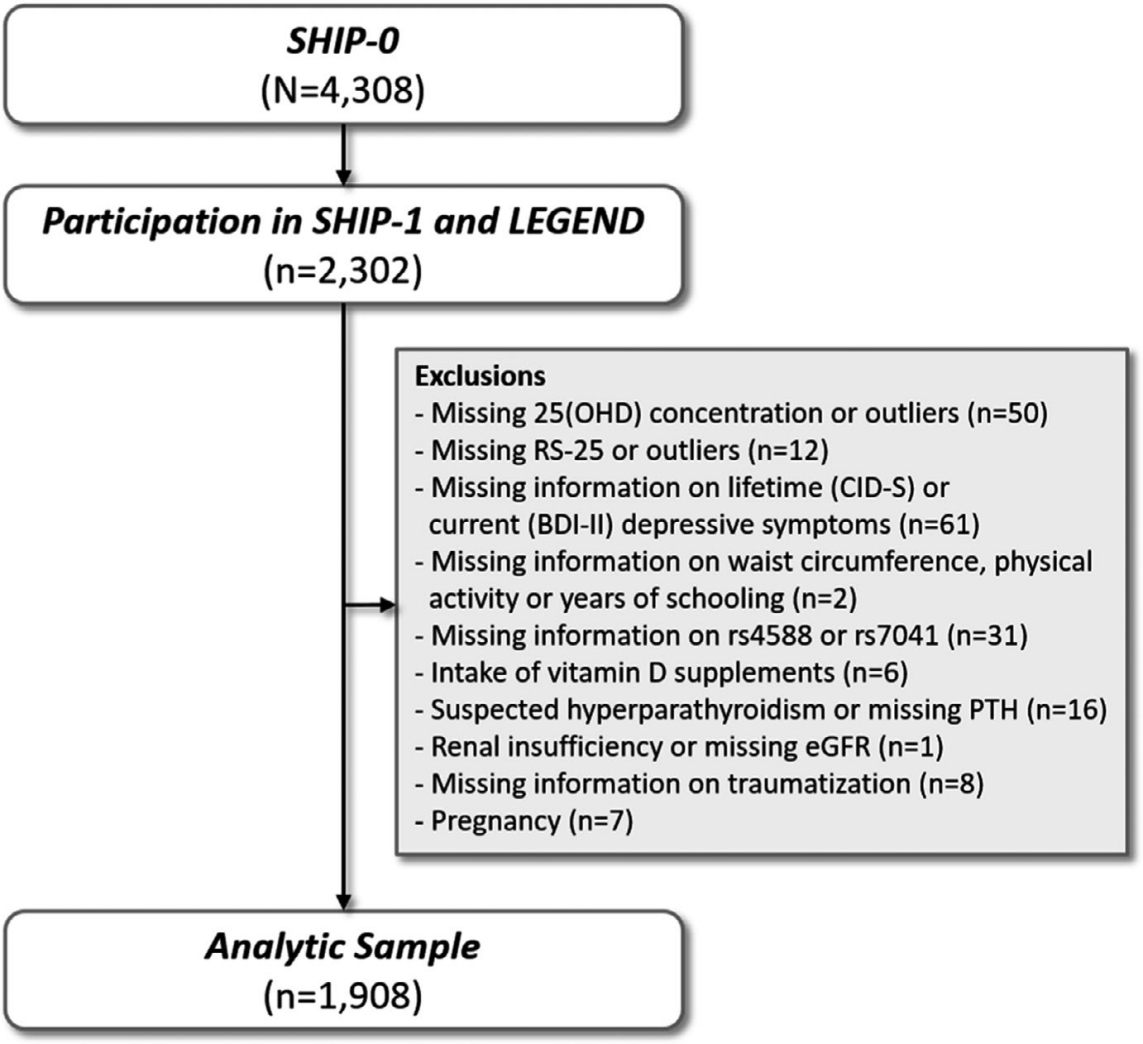
Selection of the study population. BDI‐II, Beck Depression Inventory; CID‐S, Composite International Diagnostic Screener for mental disorders; eGFR, estimated glomerular filtration rate; PTH, parathyroid hormone; RS‐25, Resilience Scale‐25; SHIP, Study of Health in Pomerania; 25(OH)D, 25‐hydroxy vitamin D

The Study of Health in Pomerania (SHIP) is a population‐based prospective cohort in northeast Germany. It is based on a representative sample from the general population that included adult men and women with German citizenship. Baseline examinations (SHIP‐0) were performed between 1997 and 2001 and included 4,308 participants aged between 20 and 79 years. The first five‐year follow‐up (SHIP‐1) was conducted between 2002 and 2006 with 3,300 participants being reexamined. Between 2007 and 2010, a psychometric assessment, named “Life‐Events and Gene‐Environment Interaction in Depression” (SHIP‐LEGENDE), was performed in consenting SHIP‐0 participants as an add‐on study. All participants of SHIP‐0 still alive at 2006 (*N* = 3,669) were invited, and 2,400 agreed to participate in SHIP‐LEGEND. In the present study, we included 2,302 SHIP‐LEGEND participants, who also underwent the SHIP‐1 examinations. From these subjects, we excluded all those with missing concentrations or outliers (mean ± 3 *SD*) in 25(OH)D concentration or RS‐25 (*n* = 62), with missing information in any of the confounding variables (*n* = 4), missing information in the SNPs (*n* = 32), intake of prescribed vitamin D supplements (*n* = 6), suspected hyperparathyroidism or missing parathyroid hormone (PTH) concentration (*n* = 16), renal insufficiency or missing eGFR (*n* = 1), missing information on traumatization (*n* = 8), missing information on BDI‐II (*n* = 57), and all pregnant women (*n* = 8). Our final analytic sample comprised 1,908 subjects. A detailed overview over the selection process is given in Figure 1.

All investigations in SHIP and SHIP‐LEGENDE were carried out in accordance with the Declaration of Helsinki, including written informed consent of all participants. The survey and study methods were approved by the institutional review boards of the University of Greifswald. Further details on SHIP, including sampling and study design, can be found elsewhere (Völzke et al., 2011).

### 2.2 Interview and physical examinations

The SHIP‐1 study program included a computer‐assisted personal interview and a large range of standardized medical examinations. Socio‐demographic characteristics and information on behavioral risk factors and medical history were collected. Subjects were asked, for example, whether they perform any physical activity. When subjects answered affirmatively they were classified as “physically active,” all other subjects as “physically inactive.” Self‐reported years of schooling were classified in <10 and ≥10 years. Adulthood traumatization was assessed using the PTSD module of the Structured Clinical Interview for Diagnostic and Statistical Manual of Mental Disorders, 4th Edition (DSM‐IV) (Elhai et al., 2008). Subjects were asked if they had been exposed to one of the following events which are enlisted as traumatic events in the DSM‐IV: combat or war‐zone experience, physical assault, rape, childhood sexual abuse, natural disaster, life‐threatening illness, serious or nearly fatal accident, imprisonment and/or torture, sudden and unexpected death of a loved one, and witnessing or learning about traumas to others. We assessed the presence of any trauma and the number of traumatic events. Depressive symptoms were determined according to the screening questions of the Composite International Diagnostic Screener for mental disorders (CID‐S) (Wittchen et al., 1999). Participants were assigned the lifetime diagnosis of depression if at least one positive answer to the two questions covering the dimensions “depressed mood” and “loss of interest” was given. During the SHIP‐1 examinations, waist circumference was measured to the nearest 0.1 cm using an inelastic tape midway between the lower rib margin and the iliac crest in the horizontal plane, with the subject standing comfortably with weight distributed evenly on both feet. Moreover, participants were asked to bring all medication taken in the last 7 days prior to the examination. The drugs were categorized according to the anatomical‐therapeutic‐chemical (ATC) classification code. The intake of prescribed vitamin D supplements was defined according to ATC A11CC.

SHIP‐LEGEND included a diagnostic interview for mental disorders based on Diagnostic and Statistical Manual for Mental Disorders (IV edition) diagnostic criteria. Socio‐demographic and clinical information were assessed by a computer‐assisted face‐to‐face interview or self‐report instruments. For psychometric assessments, various established and validated self‐report questionnaires and a newly developed interview on 80 positive and negative life events were applied. Resilience was measured applying the German version of the Resilience Scale (RS‐25) (Schumacher et al., 2005; Wagnild, 2009). It is comprised of 25 items with 7‐point Likert scale, and it is used to measure scores of five components which have been conceptualized to form a so‐called "Resilience Core" (Wagnild & Young, 1993). The components of the RS‐25 include a "purposeful life," "perseverance," "equanimity," "self‐reliance," and "the awareness of being on your own in a lot of situations in life" (Existential aloneness). Scores range from 25 to 175 with higher scores indicating a higher degree of resilience. While the original American version was comprised of the two factors "Acceptance of Self and Life" and "Personal Competence," this two factor model could not be replicated in the German version (Schumacher et al., 2005). The German RS‐25 showed good values for reliability (internal consistency Cronbach's *α* = .95, retest reliability .67–.84) and significant convergent validity with a scale for self‐efficacy expectation (*r* = .68, *p* < .001) (Schumacher et al., 2005). Current depressive symptoms were assessed using the Beck Depression Inventory (BDI‐II) (Beck et al., 1996), a 21‐item self‐report questionnaire with high reliability and validity. From the response to each of the BDI‐II items, a sum score was determined and used as continuous outcome.

### 2.3 Laboratory analyses

Nonfasting venous blood samples were obtained from all consenting participants following a standardized protocol. The single occasion samples were taken throughout the year. Season of blood sampling was defined as winter (December–February), spring (March–May), summer (June–August), or autumn (September–November). Samples were stored at −80°C in the Integrated Research Biobank of the University Medicine Greifswald and used in accordance with its regulations. Serum 25(OH)D concentrations were measured with the IDS‐iSYS 25‐Hydroxy Vitamin D chemiluminescence assay on the IDS‐iSYS Multi‐Discipline Automated Analyser (Immunodiagnostic Systems Limited). Three concentrations of control material were measured, and the coefficients of variation were 16.8%, 13.9%, and 12.0% at low, medium, and high concentrations, respectively. Subjects with 25(OH)D < 20 ng/ml were classified as vitamin D insufficient. The eGFR was calculated using the four‐variable Modification of Diet in Renal Disease formula (Levey, 2000).

### 2.4 Genetic data

In SHIP, genotyping was performed using the Affymetrix Genome‐Wide Human SNP Array 6.0 (Affymetrix). Hybridization of genomic DNA was done in accordance with the manufacturer's standard recommendations. The genetic data analysis workflow was created using the software InforSense. The overall genotyping efficiency of the GWA was 98.6%. Imputation of genotypes was performed using the HRCv1.1 reference panel and the Eagle and Minimac3 software implemented in the Michigan Imputation Server for prephasing and imputation, respectively. SNPs with a Hardy–Weinberg Equilibrium *p*‐value < .0001, a call rate < 0.95, and a MAF < 1% were removed before imputation. We specifically analyzed two common single‐nucleotide polymorphisms (SNPs) in the coding region of the Gc gene (rs4588 and rs7041). Both SNPs were imputed with imputation quality > 0.97. In rs4588, a C/A substitution leads to a Thr/Lys amino acid change, and in rs7041, a G/A substitution leads to a Glu/Asp amino acid change (Braun et al., 1992; Zhou et al., 2019). These nucleotide changes result in three commonly studied protein isoforms: Gc1S, Gc1F, and Gc2. The protein isoforms are characterized by the following haplotypes: Gc1S: rs4588 CC and rs7041 GG; Gc1F: rs4588 TT and rs7041 CC; and Gc2: rs4588 AA and rs7041 TT (Fang et al., 2009). A fourth possible homozygous haplotype (rs4588 AA and rs7041 GG) was not present in our population.

### 2.5 Statistical analyses

Characteristics of the study participants, including health‐related and genetic information, are given according to vitamin D status as medians with 1st–3rd quartiles (continuous data) or proportions (categorical data). Group differences between vitamin D insufficient and replete participants were tested for statistical significance with chi‐squared (categorical data) or Kruskal–Wallis tests (continuous data). A value of *p* < .05 was considered statistically significant.

The associations between vitamin D status or the 25(OH)D concentration and lifetime depressive symptoms as assessed by CID‐S (yes/no) were analyzed using logistic regression models, while the associations with current depressive symptoms as assessed by BDI‐II (continuous) were analyzed using Tobit regression models. Tobit regression was applied because the BDI‐II is not normally distributed with 21.2% of subjects having a value of "0." We report odds ratios with 95% confidence intervals (CI) and *p*‐values from the logistic regression models and *ß*‐coefficients with standard errors and *p*‐values from the Tobit regression models. Analyses of variance (ANOVA) and multivariable linear regression analyses were applied to assess the associations between vitamin D status (ANOVA) or the 25(OH)D concentration (linear regression) and RS‐25. We report adjusted mean RS‐25 values with 95% CIs for vitamin D insufficient and replete subjects (ANOVA). From the linear regression models, we report *ß*‐coefficients with standard errors and *p*‐values. In the above models that use the 25(OH)D concentration as continuous variable, that is, all models except from the ANOVA, 25(OH)D was log‐transformed to achieve a normal distribution of the residuals. All models were adjusted for sex, age, waist circumference, physical activity, season of blood sampling, and years of schooling. All models examining the association with RS‐25 were additionally adjusted for depressive symptoms, once for lifetime depressive symptoms and a second time for current depressive symptoms. We then tested whether sex, age, or depressive symptoms were effect modifiers (2‐way interactions) in the association between 25(OH)D and RS‐25. Moreover, we additionally adjusted the models for traumatization (yes/no) or the number of traumas and assessed whether these factors represent effect modifiers.

Subsequently, we assessed whether the Gc genotypes, rs4588 or rs7041, were associated with current depressive symptoms, log‐transformed 25(OH)D concentrations, or RS‐25. To this end, we calculated another set of multivariable linear or Tobit regression models, adjusted as mentioned above. Finally, we assessed whether statistically significant 2‐way interactions between the 25(OH)D concentration and the two SNPs or the Gc genotypes on RS‐25 were present. All statistical analyses were performed with SAS 9.4 (SAS Institute Inc.).

## 3 RESULTS

### 3.1 Health‐related characteristics

In our study population of 1908 adult subjects, 57.0% (*n* = 1,088) were vitamin D insufficient (Table 1). The majority of vitamin D insufficient subjects had provided blood samples during winter or spring (68.0%), while among the vitamin D replete subjects only 36.5% provided blood samples in these seasons. There were no statistically significant differences between vitamin D insufficient or replete participants regarding sex, age, years of schooling, current or lifetime depressive symptoms, occurrence of traumatization, or number of traumas. However, vitamin D insufficient subjects had higher waist circumferences (*p* < .001) and were more often physically inactive (*p* < .001) than vitamin D replete subjects. Moreover, we observed lower RS‐25 values in vitamin D insufficient than in replete subjects (Rs‐25:147 vs. 149, *p* = .014).

**TABLE 1:** Characteristics of the study population

Characteristics	Vitamin D insufficient (*n* = 1,088)	Vitamin D replete (*n* = 820)	*p*
Male, %	46.4	50.1	.109
Age, years	51.0 (40.0–62.0)	52.0 (40.0–62.0)	.657
Waist circumference, cm	93.1 (83.0–102.6)	89.0 (80.0–98.9)	<.001
Physically inactive, %	59.9	49.5	<.001
Depressive symptoms (CID‐S), %	17.8	16.8	.568
BDI‐II sum score	4.0 (1.0–9.0)	4.0 (1.0–9.0)	.160
Years of schooling ≥ 10, %	68.3	69.4	.608
25(OH)D, ng/ml	13.7 (10.6–16.6)	26.2 (22.5–31.7)	<.001
Season, %			<.001
Spring	39.0	19.4	
Summer	11.6	32.9	
Autumn	20.5	30.6	
Winter	29.0	17.1	
Traumatization, %	51.2	53.4	.337
*N* of traumas	1 (0–1)	1 (0–1)	.412
Gc genotypes, %			<.001
Gc1s	28.2	37.3	
Gc1f	1.84	2.44	
Gc2	10.1	5.12	
Other	59.8	55.1	
rs4588, %			<.001
AA	10.1	5.12	
AC	44.9	38.8	
CC	45.0	56.1	
rs7041, %			<.001
GG	28.2	37.3	
GT	51.1	48.5	
TT	20.8	14.2	
RS‐25	147 (135–158)	149 (137–161)	.014

Note

Data are median (1st–3rd quartile) or proportions. Group differences were tested with chi‐squared or Kruskal–Wallis tests. Subjects with 25(OH)D concentrations <20 ng/ml were defined as vitamin D insufficient, all other subjects as vitamin D replete.

Abbreviations: 25(OH)D, 25‐hydroxy vitamin D; BDI‐II, Beck Depression Inventory; RS‐25, Resilience Scale‐25.

### 3.2 Associations of the 25(OH)D concentration and lifetime (CID‐S) or current (BDI‐II) depressive symptoms

There were no associations between the 25(OH)D concentration (odds ratio 1.004, 95% confidence interval 0.753–1.339, *p* = .977) or vitamin D status (odds ratio 0.977, 95% confidence interval 0.752–1.269, *p* = .861) and lifetime depressive symptoms. In addition, there were no associations between the 25(OH)D concentration (*ß* = −0.753, *SE* = 0.438, *p* = .086) or vitamin D status (*ß* = −0.652, *SE* = 0.397, *p* = .101) and current depressive symptoms.

### 3.3 Associations of vitamin D status, the 25(OH)D concentration and RS‐25

**FIGURE 2:**
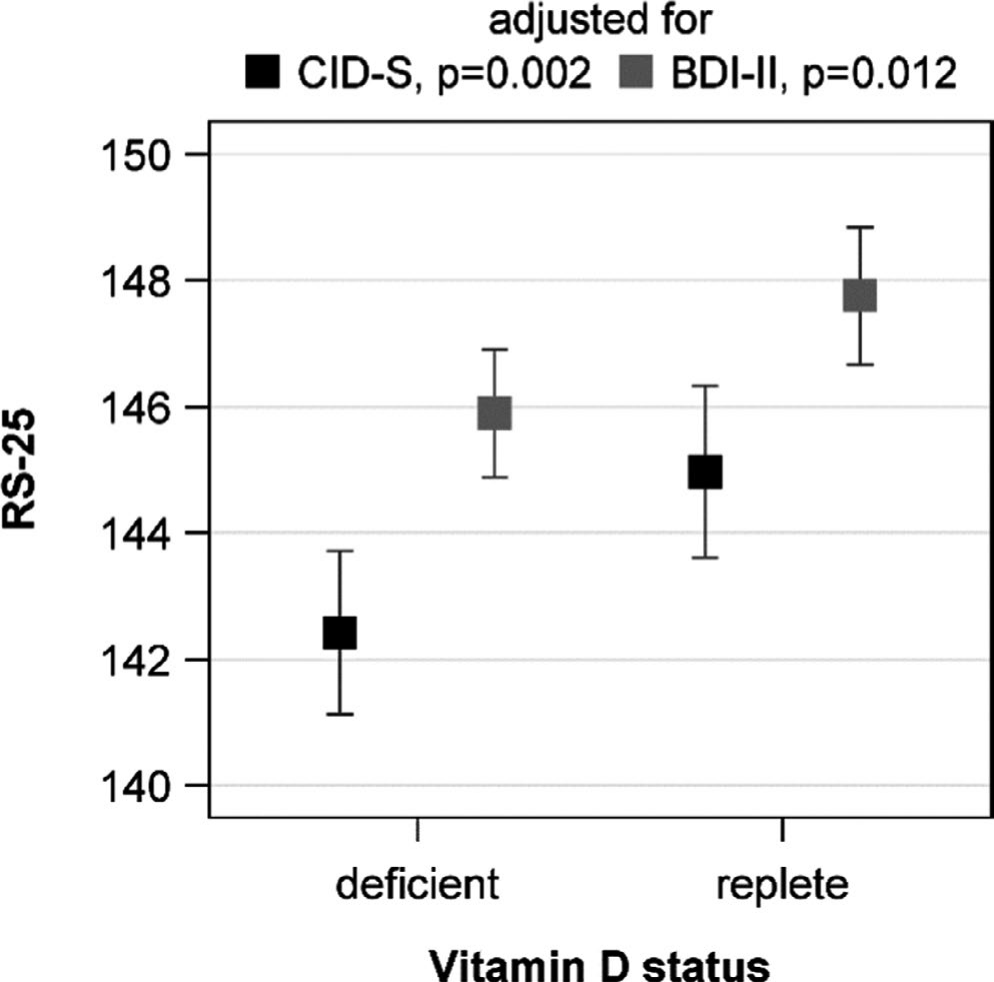
Adjusted mean RS‐25 score with 95% confidence intervals according to vitamin D status. The models were adjusted for sex, age, waist circumference, physical activity, season of blood sampling, years of schooling and lifetime (CID‐S), or current depressive symptoms (BDI‐II). Vitamin D insufficiency was defined as 25(OH)D concentrations < 20 ng/ml. BDI‐II, Beck Depression Inventory; RS‐25, Resilience Scale‐25; 25(OH)D, 25‐hydroxy vitamin D

**FIGURE 3:**
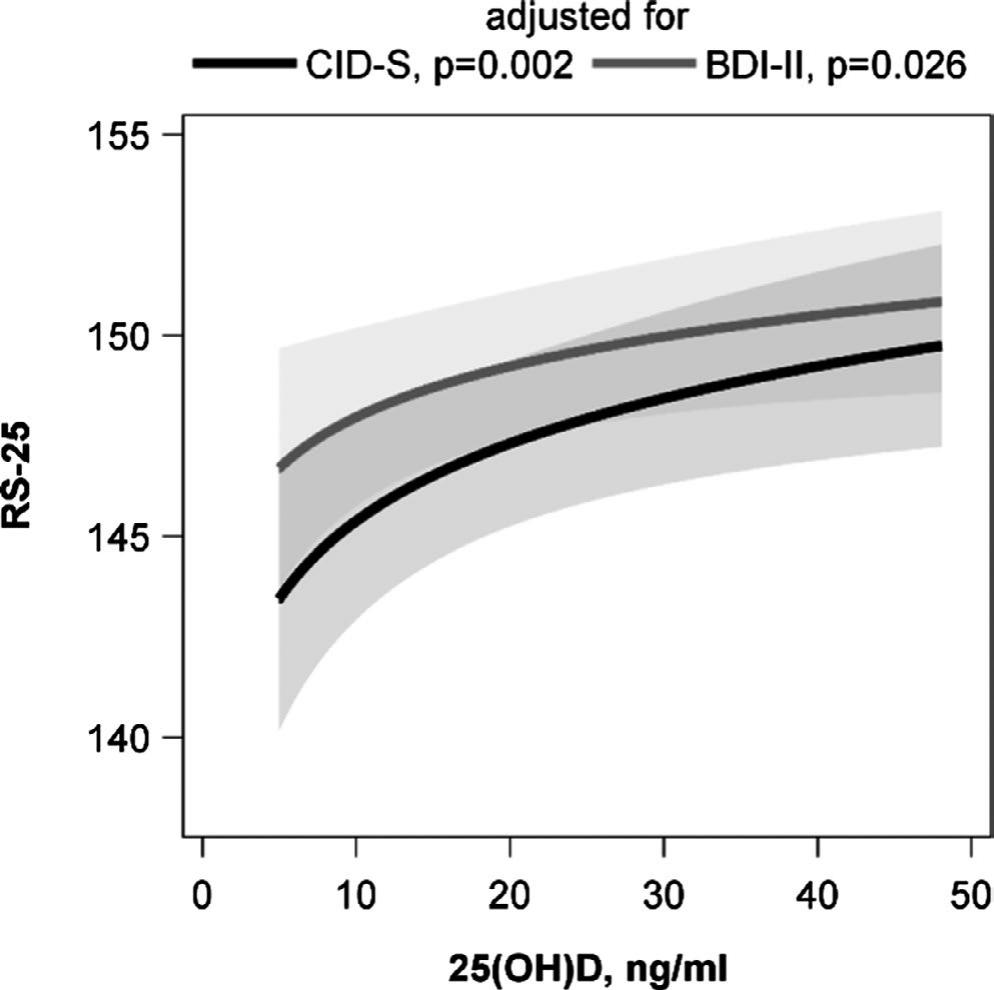
Association between the 25‐hydroxy vitamin D [25(OH)D] concentration and the RS‐25. Results from linear regression models adjusted for sex, age, waist circumference, physical activity, season of blood sampling, years of schooling and lifetime (CID‐S), or current (BDI‐II) depressive symptoms. BDI‐II, Beck Depression Inventory; RS‐25, Resilience Scale‐25

The initially observed difference in RS‐25 between vitamin D insufficient and replete subjects was confirmed in the ANOVA. Participants with 25(OH)D concentrations below 20 ng/ml had lower adjusted mean RS‐25 values than those with higher 25(OH)D concentrations. This result was independent of the adjustment for lifetime (*p* = .002) or current (*p* = .012) depressive symptoms (Figure 2). An inspection of the association with multivariable linear regression analyses revealed a positive association between the 25(OH)D concentration and RS‐25 after adjustment for lifetime depressive symptoms (*ß* = 2.782, *SE* = 0.903, *p* = .002). The adjustment for current depressive symptoms revealed a slightly attenuated association (*ß* = 1.831, *SE* = 0.819, *p* = .026) (Figure 3). These data indicate that increasing vitamin D concentrations are related to a higher resilience. Effect modification by sex, age, lifetime or current depressive symptoms, traumatization, or number of traumas was not observed (*p*‐values for all interaction terms > .050).

### 3.4 Associations of rs4588, rs7041, and the Gc Genotypes with 25(OH)D, BDI‐II, and RS‐25

**FIGURE 4:**
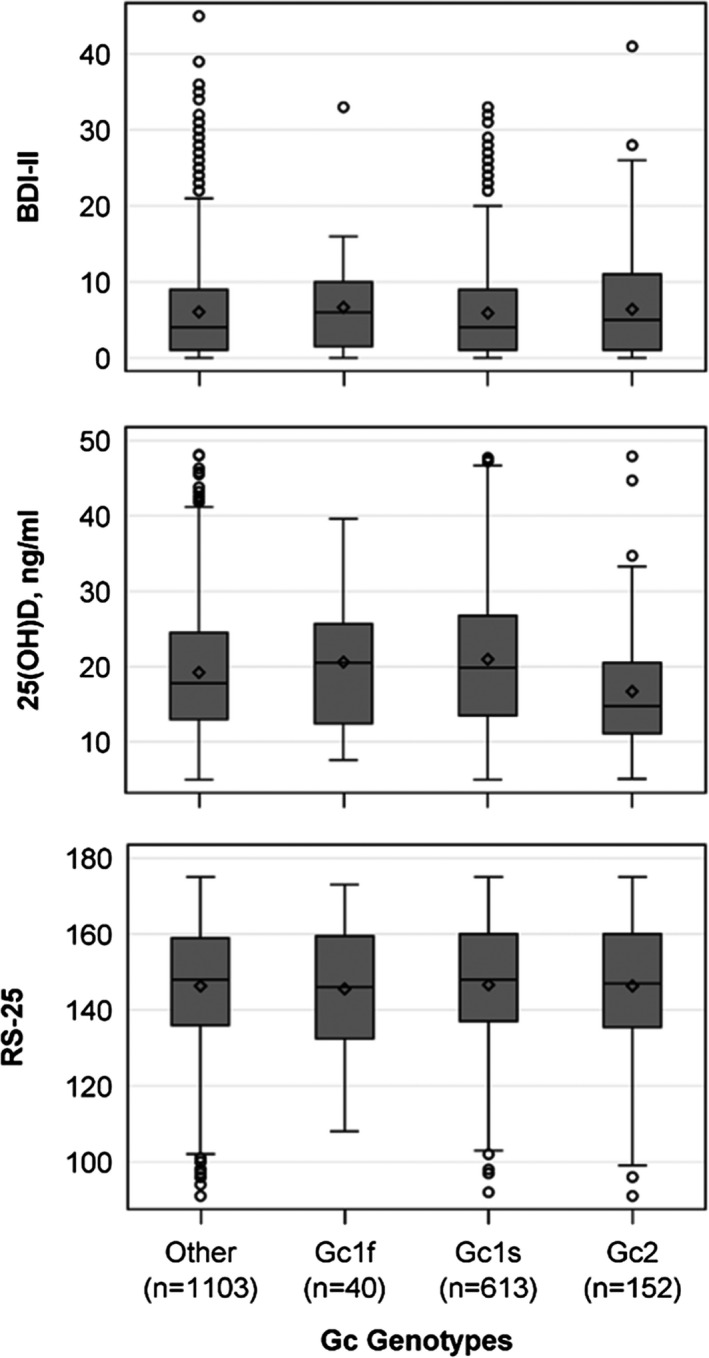
Box plots for the 25(OH)D concentration, the RS‐25, and the BDI‐II according to the Gc genotypes. BDI‐II, Beck Depression Inventory; 25(OH)D, 25‐hydroxy vitamin D; RS‐25, Resilience Scale‐25

Both polymorphisms in the vitamin D‐binding protein, rs4588 and rs7041, as well as the Gc genotypes were associated with the 25(OH)D concentration when adjusted for lifetime depressive symptoms (Table 2, Figure 4). Homozygous Gc1S carriers had significantly higher 25(OH)D concentrations and Gc2 carriers lower 25(OH)D concentrations than heterozygous subjects. Gc1F carriers, who represented only 2.1% (*n* = 40) of our study population, had 25(OH)D concentrations comparable to the heterozygous subjects. The results were almost identical when the adjustment was changed from lifetime to current depressive symptoms and are therefore not shown. Above this, there were no associations between rs4588, rs7041 and the Gc genotypes with the BDI‐II or the RS‐25 score. Also, effect modification between the SNPs or the Gc Genotypes and 25(OH)D on RS‐25 was not present in the data (*p*‐values for all interaction terms > .05).

**TABLE 2:** Associations between rs4588, rs7041, and the Gc genotypes with current depressive symptoms (BDI‐II), 25(OH)D concentrations, or the RS‐25 score

Exposure	BDI‐II^1^			25(OH)D^2^			RS‐25^2^			RS‐25^3^		
	*ß*	*SE*	*p*	*ß*	*SE*	*p*	*ß*	*SE*	*p*	*ß*	*SE*	*p*
rs4588												
AA (=reference)												
AC	−0.460	0.701	.511	0.129	0.037	<.001	0.210	1.456	.885	−0.770	1.316	.558
CC	−0.898	0.693	.195	0.193	0.036	<.001	1.048	1.438	.466	−0.317	1.301	.807
rs7041												
GG (=reference)												
GT	0.095	0.412	.818	−0.074	0.021	<.001	−0.696	0.852	.414	−0.559	0.770	.468
TT	0.590	0.536	.271	−0.134	0.028	<.001	−0.428	1.112	.700	0.652	1.004	.516
Gc geno‐types												
Other (=reference)												
Gc1s	0.880	1.263	.486	0.074	0.021	<.001	0.524	0.829	.527	0.301	0.750	.688
Gc1f	−0.118	0.401	.768	0.032	0.067	.629	−1.356	2.647	.608	−0.390	2.394	.871
Gc2	0.677	0.686	.324	−0.137	0.036	<.001	−0.513	1.425	.719	0.622	1.288	.629

Note

Results from linear [25(OH)D and RS‐25] or Tobit (BDI‐II) regression models adjusted for ^1^sex, age, waist circumference, physical activity, season of blood sampling, and years of schooling, ^2^plus lifetime depressive symptoms (CID‐S), or ^3^current depressive symptoms (BDI‐II).

Abbreviations: 25(OH)D, 25‐hydroxy vitamin DBDI‐II, Beck Depression Inventory; CID‐S, Composite International Diagnostic Screener for mental disorders; RS‐25, Resilience Scale‐25; *SE*, standard error.

## 4 DISCUSSION

Finding strong associations between vitamin D concentrations and resilience fits well with various previous findings of a relation between vitamin D with different mental health conditions (Chiang et al., 2016; Kamal et al., 2014; Terock et al., 2020). Given that resilience is considered to reflect generally diminished vulnerability to life stressors, our findings support the concept that low vitamin D levels represent a broad risk factor to different stress‐related psychiatric conditions like depression and PTSD. Different mechanisms of how vitamin D impacts on stress reactivity and pathophysiology of psychiatric disorders have been proposed: After activation of 25(OH)D to 1,25(OH)D by the 1α‐hydroxylase, vitamin D binds to intracellular vitamin D receptors (Holick, 2007), which subsequently occupy vitamin D response elements around promotor regions of different genes (Uitterlinden et al., 2004). Specifically, different genes involved in the regulation the serotonin metabolism, which is considered to take in a key role in the pathophysiology of conditions like depression, PTSD, and anxiety disorders, were found to be influenced by vitamin D levels. For example, vitamin D was reported to activate the transcription of the tryptophan hydroxylase 2 (TPH2) gene (Kaneko et al., 2015; Patrick & Ames, 2014) and the tyrosine hydroxylase gene (Puchacz et al., 1996). The tryptophan hydroxylase is considered the rate‐limiting enzyme for the serotonin synthesis with TPH2 being the corresponding gene primarily expressed in the brain (Walther et al., 2003). Functional polymorphisms of the TPH2 gene have repeatedly been found to be associated with depressive disorders (Gao et al., 2012; Zill et al., 2004), PTSD (Goenjian et al., 2012, 2015), and the personality trait harm avoidance (Reuter et al., 2007) which is negatively associated with resilience (Kim et al., 2013; Simeon et al., 2007). Likewise, vitamin D has been reported to influence the expression of the tyrosine hydroxylase gene in the adrenal gland (Puchacz et al., 1996). The tyrosine hydroxylase takes in a key role in the synthesis of catecholamines, that is, dopamine, adrenaline, and noradrenaline, which have also been linked with stress‐related psychiatric disorders and resilience (Krystal & Neumeister, 2009). Results from other studies suggest that vitamin D interacts with the neuroendocrine stress response, particularly with the hypothalamic–pituitary–adrenal (HPA)axis: For example, Obradovic et al. (2006) reported that 1,25(OH)D could reverse the inhibiting effects of glucocorticoids hippocampal neurite cell growth and differentiation. Likewise, Jiang et al. (2013) found that in rats showing a depressive‐like state after exposure to chronic stress, genes involved in the vitamin D metabolism and local vitamin D levels were upregulated, indicating that vitamin D is involved in the processes protecting the brain from the deteriorating effects of chronic stress.

However, given that our data are based on a cross‐sectional study, no causal conclusions can be drawn and it is well conceivable that poor resilience leads to reduced vitamin D levels due to unbeneficial health‐related behavior. In fact, there is evidence showing that psychological resilience and resilience promoting factors are associated with lifestyle factors including aspects of nutrition and physical activity (Mistry et al., 2009; Shin & Kang, 2015). Also, psychiatric conditions in general and particularly depressive disorders, which are related to low resilience, were found to be associated with health‐related behaviors which may lead to low vitamin D levels like physical inactivity and poor diet (Skrove et al., 2013). Still, all analyses in this study were adjusted for different lifestyle factors including physical activity, season, and waist circumference. Moreover, additional adjustment for the lifetime diagnosis of depression and current depressive symptoms only slightly attenuated the observed associations. Finally, although traumatization is important risk factor for poor resilience (Bonanno, 2005; Terock et al., 2019), no interaction between status of traumatization or number of traumatic events with resilience on vitamin D levels was found, indicating that the relationship between resilience and vitamin D levels is independent from putative effects of traumatization on lifestyle. Still, detailed information about nutrition habits or sunlight exposure was not available and may well have contributed to our results.

To further elucidate the question of causality for the association between resilience and vitamin D levels, we analyzed genetic variables with impact on the vitamin D metabolism independently from behavioral or clinical factors. While, in consistence with previous studies (Bikle & Gee, 1989), rs4588 and rs7041 were highly associated with vitamin D levels, we did not find significant associations of the polymorphisms and the corresponding haplotypes with resilience or depression, suggesting that our results are at least partly attributable to lifestyle factors. Still, since low vitamin D levels were found to predispose to a wide range of physical conditions including hypertension, diabetes, metabolic syndrome, congestive heart failure, and chronic vascular inflammation (Holick, 2007; Zittermann, 2006) and that vitamin D insufficiency is a risk factor for fatal outcomes of these conditions over and above of the general health status (Schöttker & Brenner, 2015), our findings may also contribute to explain the association of resilience with poor physical health and even increased mortality (Stewart & Yuen, 2011; Surtees et al., 2006).

Finding no association between vitamin D concentrations and depressive symptoms or the lifetime diagnosis of depression adds to the currently inconclusive body of evidence with some studies showing associations (Armstrong et al., 2007; Goltz et al., 2017; Milaneschi et al., 2014), while other authors did not find evidence for such a relationship (Parker & Brotchie, 2011). However, findings from longitudinal studies did not suggest that vitamin D influences onset and course of depressive symptoms (Jovanova et al., 2017; van den Berg et al., 2016). In their systematic review, Autier et al. (2014) evaluated existing observational and interventional studies on the effects of 25(OH)D on various somatic disorders (Autier et al., 2014). The authors concluded that the discrepancy between the high number of positive association in cross‐sectional studies and the weak evidence for effects of vitamin D in interventional studies indicates that low 25(OH)D levels are a marker of ill health, for example, due to inflammatory processes leading to a reduction of vitamin D levels. In the context of these results, the lack of an association between vitamin D, Gc genotypes, and depression in this study calls a causal role of vitamin D in depression further into question. However, previous studies revealed effects of low vitamin D on increased mortality in older depressed subjects (van den Berg et al., 2016) indicating that low vitamin D may be a relevant problem in depressed subjects as it may contribute to the higher mortality associated with depression.

Our study has several strengths including the large and well‐characterized community‐based sample, the adjustment for various potential confounders, and the use of well‐established psychometric measures. However, some limitations need to be acknowledged: First, as noted above, due to the cross‐sectional design of our study the direction of causality for the observed association between 25(OH)D levels and resilience cannot be clearly determined. Our results were controlled for several confounders including sex, age, waist circumference, physical inactivity, season, and depressive symptoms, thus reducing the influence of lifestyle factors. Considering the lack of association between Gc gene polymorphisms and resilience, our findings suggest a contribution of behavioral and clinical factors to the association. Still, prospective studies are needed in order to disentangle predictor from outcome. Second, Gc levels were not measured in this study. Although the tested genotypes were previously found to explain a substantial proportion of the variance of Gc levels and affinity (Arnaud & Constans, 1993; Lauridsen et al., 2005), other factors including genetic polymorphisms not tested in this study also contribute to variability in Gc plasma levels. Future studies should therefore directly investigate circulating Gc levels. Third, unfortunately, the variable physical activity only roughly splits between active and inactive participants which limits the informative value of this important factor in our study. More specific information on the type and duration of physical activity would be helpful to better assess the effects of lifestyle factors on the observed association.

In summary, this study provides first evidence for a highly significant association between psychological resilience and low vitamin D levels over and above the effects of various behavioral and metabolic as well as seasonal confounders. Importantly, our results remained largely stable after additional adjustment for depression. With respect to the role of resilience as a broad protective factor for the development of stress‐related psychiatric and physical disorders, these findings may contribute to explain the converging evidence for the role of vitamin D metabolism in the pathogenesis of different health conditions.

## 5 CONCLUSION

Our results support the concept of low vitamin D as a general risk factor to stress‐related psychopathologies, while our findings do not suggest that altered vitamin D levels are specifically linked with depression.

Moreover, given that we did not identify associations between genotypes of the Gc gene and resilience, our findings indicate that health‐related behavior contributed to the observed association between vitamin D levels and resilience. Although our results were adjusted for various potential confounders, insufficient operationalization of variables and health factors not covered by our confounder set may have been involved. Our findings showing associations of vitamin D levels with resilience could help to explain the relationship between low resilience and poor physical health.

## CONFLICT OF INTEREST

All authors declare no conflicts of interests related to this work.

## AUTHOR CONTRIBUTION

JT, AH, DJ, JM, and HJG developed the content alignment of manuscript. AH calculated the analyses. JT and AH wrote the manuscript with support of DJ and AM. HV raised the funding, and implemented and supervised the SHIP studies. HJG implemented and supervised the use of the TAS‐20. All authors have read and approved the final version of the manuscript.

### Peer Review

The peer review history for this article is available at https://publons.com/publon/10.1002/brb3.1884.

## Data Availability

The data that support the findings of this study are available from the corresponding author upon reasonable request.
